# Ten-year decrease of acquired methicillin-resistant Staphylococcus aureus (MRSA) bacteremia at a single institution: the result of a multifaceted program combining cross-transmission prevention and antimicrobial stewardship

**DOI:** 10.1186/2047-2994-1-18

**Published:** 2012-05-18

**Authors:** Annie Chalfine, Marie-Dominique Kitzis, Yvonnick Bezie, Adel Benali, Laurence Perniceni, Jean-Claude Nguyen, Marie Françoise Dumay, Jacqueline Gonot, Gilles Rejasse, Fred Goldstein, Jean Carlet, Benoît Misset

**Affiliations:** 1 Infection Control Committee, Groupe hospitalier Paris Saint Joseph, Paris, France; 2 Infection Control Unit, Groupe hospitalier Paris Saint Joseph, Paris, France; 3 Clinical Microbiology Unit, Groupe hospitalier Paris Saint Joseph, Paris, France; 4 Pharmacy, Groupe hospitalier Paris Saint Joseph, Paris, France; 5 Infectious Disease Team, Groupe hospitalier Paris Saint Joseph, Paris, France; 6 Medical Information Unit, Groupe hospitalier Paris Saint Joseph, Paris, France; 7 Intensive Care Unit, Groupe hospitalier Paris Saint Joseph, Paris, France; 8 Faculté de Médecine, Paris Sorbonne Cité, Université Paris-Descartes, Paris, France

**Keywords:** MRSA, Bacteremia, Hospital-acquired, Isolation precaution, Alcohol based hand rub, Antibiotic stewardship

## Abstract

**Background:**

In France, the proportion of MRSA has been over 25% since 2000. Prevention of hospital-acquired (HA) MRSA spread is based on isolation precautions and antibiotic stewardship. At our institution, before 2000, the Infection Disease and the Infection Control teams had failed to reduce HA-MRSA rates.

**Objectives and methods:**

We implemented a multifaceted hospital-wide prevention program and measured the effects on HA-MRSA colonization and bacteremia rates between 2000 and 2009. From 2000 to 2003, active screening and decontamination of ICU patients, hospital wide alcohol based hand rubs (ABHR) use, control of specific classes of antibiotics, compliance audits, and feed-backs to the care providers were successively implemented. The efficacy of the program was assessed by HA-MRSA colonized and bacteremic patient rates per 1000 patient-days in patients hospitalized for more than twenty-four hours.

**Results:**

Compliance with the isolation practices increased between 2000 and 2009. Consumption of ABHR increased from 6.8 L to 27.5 L per 1000 patient-days. The use of antibiotic Defined Daily Doses (DDD) per 1000 patient-days decreased by 31%. HA-MRSA colonization decreased by 84% from 1.09 to 0.17 per 1000 patient-days and HA-MRSA bacteremia by 93%, from 0.15 to 0.01 per 1000 patient-days (*p* < 10^−7^ for each rate).

**Conclusions:**

In an area highly endemic for MRSA, a multifaceted prevention program allows for sustainable reduction in HA-MRSA bacteremia rates.

## Introduction

Methicillin-resistant *Staphylococcus aureus* (MRSA) has become a nosocomial pathogen worldwide [1-3]. The European Antimicrobial Resistance Surveillance System (EARSS) has documented that MRSA represents more than 25% of the SA strains in France since 2000 [4]. MRSA bacteremia is associated with an approximate mortality rate of 50% [5]. Factors contributing to MRSA bacteremia include the use of invasive devices, concentration of very sick patients, high work-load, microorganisms cross-transmission, and widespread use of antibiotic therapy [6]. To improve this situation, current recommendations are based on two approaches. The first relies on barrier precautions, hand hygiene and environmental cleaning for MRSA patients. The second is to decrease the antibiotic pressure by controlling antibiotic prescribing [6]. We implemented a program based on preventive measures of cross-transmission and on antibiotic stewardship, associated with compliance assessments and feed-back of the results to the care providers. We report the impact of this program on the rates of HA-MRSA bacteremia from January 2000 to December 2009.

## Methods

### Setting, case-mix and local background

**Figure 1:**
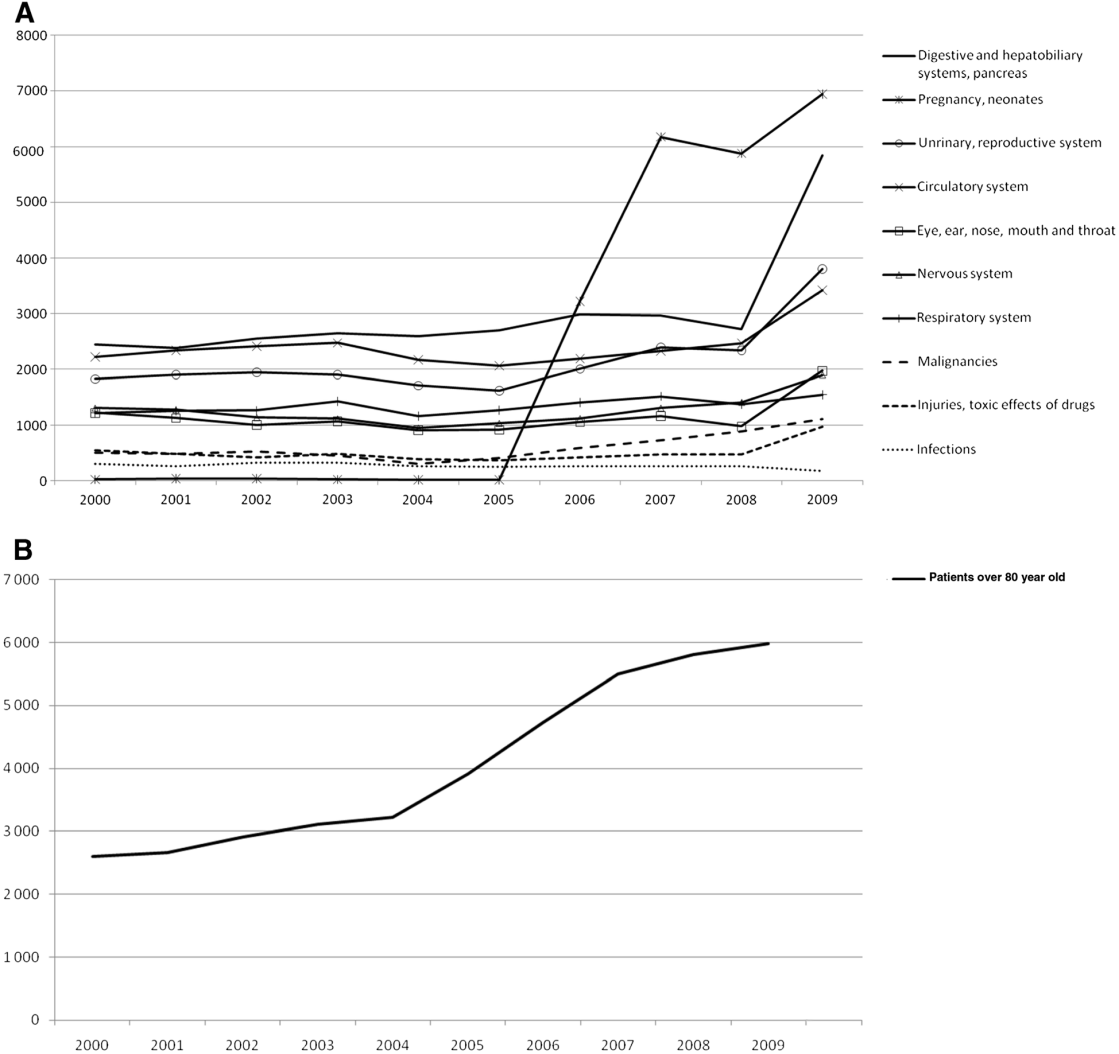
**Evolution of the case-mix of the Saint-Joseph hospital over time.** Legend: X axis: years. Y axis: number of patients admitted per year. **A:** The case-mix is described with major diagnostic categories, derived from the diagnosed related group (DRG) system used in France. The appearance of cases of pregnancy and neonates is due to the implementation of these activities in 2006. The major increase in digestive cases is due to the implementation of a new activity of proctology in 2009. **B:** The increase in patient over 80 year old is due to the enlargement of the geriatric unit in 2006.

The Saint-Joseph hospital is a private nonprofit hospital, serving as a primary and tertiary care facility in Paris. It has been running 450 adult care beds before 2006, and 540 beds since 2006, after merging with two hospitals of the same area. This merge induced the implementation of three new activities: obstetrics and geriatrics from 2006, and proctology from 2009. Thus, case-mix changed, as assessed with the major diagnostic categories of the North-American derived French DRG system prior activities were unchanged or increased, and the new activities increased markedly (Figure 1). The hospital includes two 10-bed Intensive Care Units (ICU). The Infection Disease (ID) team, introduced in 1980, has been giving advice to physicians in the wards for patients with a positive microbiology result. Since 1993, the Infection Control Unit implemented an alert system for patients colonized with multi-resistant bacteria. Despite these measures, the prevalence of MRSA colonization had reached a high endemicity. By 1999, 0.94% of all admitted patients were colonized with MRSA and MRSA acquisitions reached 1.06 per 1000 patient-days.

### Prevention of cross-transmission

A program based on international recommendations [6] was elaborated by the Infection Control Committee (ICC). Preventive measures of cross-transmission were updated each year since 1999. The clinician prescribes microbiological samples according to the status of the patient. Identification of MRSA is based on clinical samples in the wards and on active screening in the ICU [7], made upon admission and weekly, using BBL-CHROMagar (BD Diagnostics, Heidelberg, Germany) from 1999 and BD GeneOhm (MRSA) real-time PCR assay (BD Diagnostics, San Diego, CA, USA) from 2003 [8]. Colonized patients are placed in contact isolation. Training sessions for contact isolation have been carried out twice a year in each unit since 2000. Barrier precautions include the use of single rooms [9], gowns for contact with the patient, and gloves for contact with biological fluids. Room cleaning is performed twice a day [10]. Disinfectant wipes are used for disinfection of small items, including stethoscopes [11]. Alcoholic based hand rubbing (ABHR) replaced hand washing in 2000. Indications for ABHR are: [1] before and [2] after contact with the patient, [3] between two cares of the same patient, [4] after contact with patient’s close environment, and [5] before any care events using aseptic techniques [12]. ABHR dispensers are available in patient’s room, on nurse’s trolley and for individual use. Since 1999 in the ICU, decolonization of MRSA carriers includes daily bathing with a povidone iodine antiseptic soap [13], and three times daily nasal mupirocin during five days [14].

Within 48 hours following the colonization, the Infection Control Unit assesses compliance with the isolation measures. Since 2002, this unit audits twice a year MRSA signaling, presence of ABHR, of gowns and of disinfectant wipes in rooms.

### Antibiotic stewardship

Once a year, the Pharmacy reports the evolution of antibiotic Defined Daily Doses (DDD) per 1000 patient-days to the ICC. Since 2002, in view of a shift of prescriptions towards broad-spectrum antibiotics, the pharmacy provides daily the list of the patients on antibiotics. The ID team has to visit all the patients on antibiotics, except surgical prophylaxis, for adjustments of antibiotic prescribing.

### MRSA rates

We included all patients admitted to hospital for at least 24 hours. Colonization was defined as isolation of MRSA from any anatomical site, and bacteremia as isolation of MRSA from a blood culture. Colonization and bacteremia were classified as “hospital acquired” (HA) if the first positive sample was taken after 48 hours of admission. HA-MRSA colonized and bacteremic patients were reported as incidence density rates per 1000 patient-days. These rates were presented four times a year to the hospital managers.

### Ethical issues

The program underwent ethical review at the institutional review board of the Saint-Joseph hospital and was considered an epidemiological activity which did not require patient consent to participate.

### Statistical analysis

Changes in annual incidence rates of MRSA colonized and/or bacteremic patients and antibiotic usage over the study period were analyzed using the chi square test for trends. The different interventions were unlikely to affect data collection as sources and methods were the same before and after the interventions, the outcome variables were shown to the care providers but were objective ones and considered reliable, all the patients of the hospital were similarly assessed during the study period. The link between annual ABHR use and HA-MRSA rates was assessed with the Spearman’s rank correlation coefficient. A *p* value <0.05 was considered statistically significant.

## Results

### Patients

The number of admitted patients for more than 24 hours was 171 366 during the 10 year period, representing 1 290 865 patient-days. The annual number of patients increased from 12 403 in 2000 to 24 027 in 2009 due to an increase in bed capacity in 2006 and a reduction in mean length of stay, from 9.13 days in 2000 to 6.38 days in 2009. The annual number of surgical procedures increased from 6 554 in 2000 to 9 647 in 2009 (Table 1).

**Table 1:** Description of the patient population admitted to the hospital

	**2000**	**2001**	**2002**	**2003**	**2004**	**2005**	**2006**	**2007**	**2008**	**2009**
Patients admitted for >24 h										
Annual number of patient admissions	12 403	12 332	12 842	13 150	13 328	13 409	19 934	25 308	24 633	24 027
Annual number of patient-days	113 194	112 547	111 073	104 601	104 829	105 734	144 021	174 141	167 442	153 283
Average length of stays (days)	9.13	9.13	8.65	7.95	7.87	7.89	7.22	6.88	6.80	6.38
Mean age (years)	63.0	62.7	61.8	61.6	63.0	63.0	60.0	57.9	59.1	59.9
Annual number of surgical procedures	6 554	6 561	7 126	7 182	7 283	7 413	8 066	9 888	9 328	9 647
Annual number of patients admitted to the ICU	339	383	344	404	401	453	465	347	342	418

### Adherence to preventive measures

**Figure 2:**
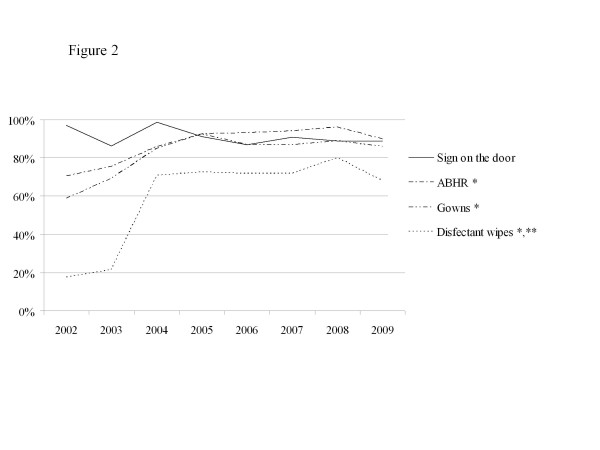
**Surrogates of compliance with isolation precautions.** Legend: X axis: years. Y axis: percentage of accurate presence of each items during audits. *: Must be present in MRSA patient’s room. **: Disinfectant wipes were provided for decontamination of small items such as stethoscopes or portable Doppler probes.

ABHR use increased from 6.8 L per 1000 patient-days in 2000 to 27.5 L in 2009 and was inversely proportional to the incidence of HA-MRSA colonized patients (r^2^ = 0.94, *p* < 10^−3^). Isolation practice determinants were assessed in 865 patients among 1 380 (63%) identified as colonized with MRSA. Of these, 605 (70%) were hospitalized in a single room. Compliance was close to 90% for the presence of the sign on the patient’s door, and ABHR and gowns in the room. The presence of disinfectant wipes increased to 70% after 2 years of audits (Figure 2).

### Antibiotic stewardship

**Figure 3:**
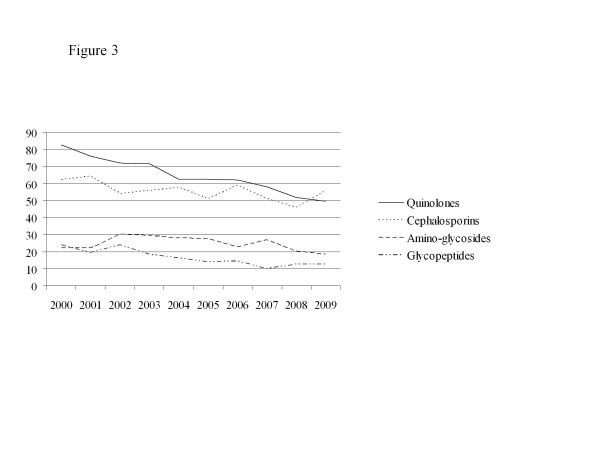
**Use of quinolones, cephalosporins, aminoglycosides and glycopeptides over time.** Legend: X axis: years. Y axis: defined daily dose (DDD) per 1000 patient-days. Chi^2^ test for trends: *p* < 10^−7^ for each class of antibiotics.

From 2003 to 2009, the ID team carried out 25 328 formal consults for 20 262 patients. The annual use of antibiotics decreased by 31% from 812.4 to 561.8 DDD per 1000 patient-days between 2000 and 2009 (*p* < 10^−7^). The use of penicillins, cephalosporins, aminoglycosides, quinolones, and glycopeptides decreased by 26%, 10%, 17%, 40% and 47% respectively (*p* < 10^−7^ for each class) (Figure 3).

### Incidence rates

**Figure 4:**
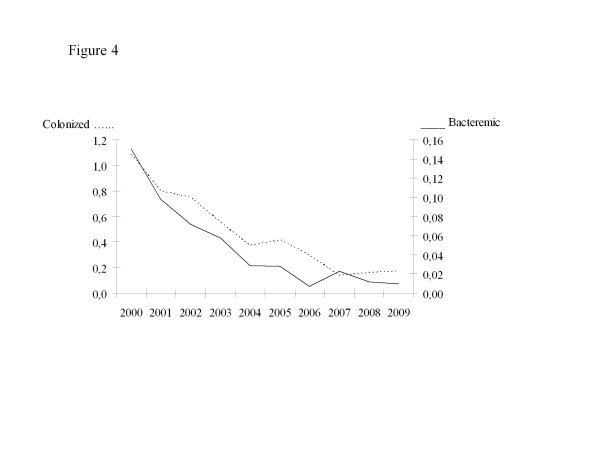
**Hospital-acquired MRSA (HA-MRSA) per 1000 patient-days over time.** Legend: X axis: years. Colonized patients are represented as dotted line and bacteremic patients as solid line. Left Y axis: number of HA-MRSA colonized patients per 1000 patient-days. Right Y axis: number of HA-MRSA bacteremic patients per 1000 patient-days. Chi^2^ test for trends: *p* < 10^−7^ for both colonized and bacteremic patients.

**Figure 5:**
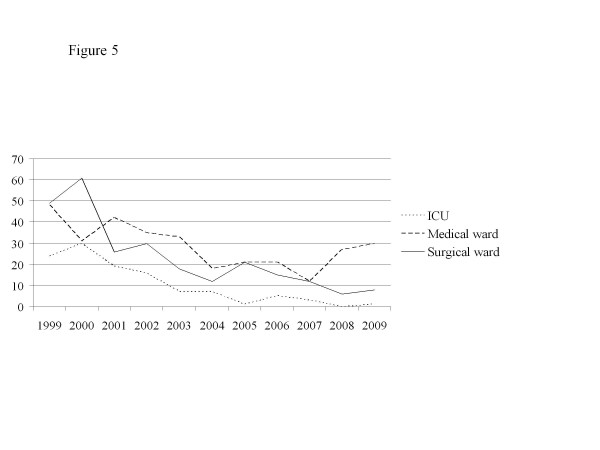
**Annual new cases of HA-MRSA colonized patients in ICU, medical wards and surgical wards.** Legend: X axis: years. Y axis: Crude number of new cases.

We documented 1 380 MRSA colonized patients including 557 HA cases (40.3%), and 122 MRSA blood stream infections (BSI) including 56 HA-BSI cases (45.9%). Annual rates of HA-MRSA colonized patients decreased by 84%, from 1.09 to 0.17 per 1000 patient-days (*p* < 10^−7^). Annual rates HA-MRSA bacteremic patients decreased by 93% from 0.15 to 0.01 per 1000 patient-days (*p* < 10^−7^) (Figure 4). The numbers of HA-MRSA bacteremic patients decreased in all the categories of wards of the hospital (Figure 5).

## Discussion

Despite many attempts to combat MRSA spread, MRSA is rapidly becoming endemic worldwide [3]. The long term impact of an institutional program had not been documented in the literature. In this study, we observed a profound and sustained reduction in HA-MRSA bacteremia by implementing a program based on cross-transmission prevention measures and antibiotic stewardship. This reduction occurred while the endemicity of MRSA in France was among the highest in Europe [4].

MRSA bacteremia is a consequence of prior colonization [5]. Prevention of MRSA bacteremia must target cross-infection and the use of antibiotics which select resistant strains to methicillin [15]. Contact precautions had an impact on the MRSA patient reservoir and the rate of nosocomial MRSA bacteremia during an epidemic outbreak [16]. A reduction in MRSA bacteremia was observed when isolation of ICU patients was based on active screening [17]. Isolation in single rooms was successful in the ICU [17] but not in peripheral wards [9]. A program based on barrier precautions was able to reduce the proportion of MRSA among *S. aureus* strains in a French hospital network [18].

Several aspects of our program may have contributed to the reduction of MRSA colonization and MRSA related healthcare infections. This interventional program was a long term strategy spanning and included regular educational events, prescribing interventions and continuous professional education at all healthcare worker levels. We observed an increase in the compliance of the care providers to isolation measures. Annual consumption of ABHR was correlated with the decrease in HA-MRSA rates. We observed a decrease in both MRSA colonization and MRSA BSI rates, consistent with the fact that prior MRSA colonization precedes infection. A progressive reduction of the MRSA reservoir size may have resulted in a reduction in the MRSA colonization pressure [19]. To limit the emergence of multi-resistant microorganisms [15], the ID team was dedicated to give advice and reduce inappropriate use of antibiotics. Antibiotic use decreased by 31% in 10 years and the most important decrease was observed for quinolones and glycopeptides. In fact, quinolone use was associated with MRSA isolation among hospitalized patients [20] and patients previously exposed to quinolones are at higher risk of acquiring MRSA [21]. By contrast to other antibiotics, we attribute the decrease in glycopeptide use to a consequence of the reduction in MRSA infections.

Our study has several limitations. First, we used a before-after design [22], so the decrease we observed may have been due to a change in MRSA epidemiology outside the hospital. Yet, the EARSS network shows that the rate of MRSA remained over 25% in France until 2007 [4]. We could not perform a time series analysis because the intervention we implemented was a multifaceted program which components started at different times, depending on the evolution of guidelines and new information from the medical literature. Therefore, we could not determine a specific point in time when either intervention occurred for the entire hospital nor assess a sufficient number of observations between each intervention to perform time series analysis [23].

Second, our hospital case-mix changed in 2006, when 90 acute care beds were added to the hospital. This change in case-mix may have had an impact on MRSA epidemiology. The increase in patients without much co-morbidity (i.e. pregnant women) or with short hospital stay (proctology procedures) may have reduced the risk for MRSA cross-transmission while the increase in elderly people may have increased this risk. Also, our data shows an increasing number of surgical interventions and patients admitted to the ICU, reflecting a sustained level of patient severity and a risk factor for cross-transmission, HA-infections and antibiotic consumption. Outside the ICU, we consider that the risk of cross-transmission is higher in geriatric and vascular patients, due to their relatively frequent chronic portage of MRSA and/or open wounds. These populations have increased during the study period. Additionally, the decrease in MRSA bacteremia had begun largely before 2006. Therefore we estimate it unlikely that the reduction in MRSA cross-transmission was substantially induced by the change in case-mix we observed.

Third, active screening was not performed outside the ICU. This was intentional [24] but may have led to missing cases imported from the community and overestimated the number of HA-MRSA. However, we observed a similar decrease in HA-MRSA rates in ICU patients. Fourth, the resistance and virulence of MRSA strains may have changed over the years. MRSA strains in the Paris area have become more frequently sensitive to gentamicin since 1990, a fact which was either due to a reduction in the use of gentamicin or to the introduction of various strains from outside the hospital [25]. This resistance profile has not changed substantially since 1995. An epidemic strain of Glycopeptide Intermediate SA (GISA) [26] was responsible for 9 cases of bacteremia during the year 2000. Recently, considerable differences in the genetic diversity of MRSA were documented between European countries [27], suggesting that outbreaks play an important role in MRSA epidemiology. Finally, the cost of our program was not assessed. The reduction of MRSA bacteremia and antibiotic consumption we observed were the main benefits for our patients at both the individual and collective levels. The cost of MRSA nosocomial infections such as pneumonia is high [28] and a continuous quality improvement (CQI) program was cost-effective [29] but the respective roles of each aspect of a multifaceted program remain to be assessed [30].

## Conclusion

In an area endemic for MRSA, a program combining several strategies aimed at reducing cross-transmission and antibiotic selection pressure allowed for a profound and sustained decrease in HA-MRSA bacteremia. The multifaceted aspect of our program was considered essential to its success. Future studies should address the role of SA genetic diversity in MRSA endemics.

## Competing interest

The authors have no personal or financial conflicts of interest to declare.

## Authors’ contribution

AC, AB, MFD, JG, JC and BM designed the program. AC, LP and JG implemented the prevention interventions and performed audits. MDK, JCN and FWG performed microbiological detection and analyses. GR provided case-mix assessment. AC, JC and BM drafted the manuscript. All authors read and approved the final manuscript.

## Financial support

None.
